# Supplementary material for: A SLC4 family bicarbonate transporter is critical for intracellular pH regulation and biomineralization in sea urchin embryos
Source: eLife. 2018 May 1;7:e36600. doi: 10.7554/eLife.36600 (PMC5986267; doi:10.7554/eLife.36600)
Supplement: Supplementary file 1. — Table S2: pH regulatory parameters of primary mesenchyme cells in control and SpSlc4a10 morphants along the ammonia pulse experiment. Intracellular buffercapacitiy (ß) was calculated using the equation: ß = ∆[NH4+]/∆pHi. Table S3: Seawater physico-chemical parameters monitored during the pH manipulation experiment. Parameters measured include Salinity, Temperature, pH (NBS scale), and total dissolved inorganic carbon (CT). pHNBS and CT were used to calculate the carbonate system, including pCO2, total alkalinity (AT) and the satuation states for calcite (ΩCa) and aragonite (ΩAr). Table S4: List of primers used for molecular cloning. Table S5 List of primers used for qPCR. [file elife-36600-supp1.doc]

**Supplemental material to:**

**A SLC4 family bicarbonate transporter regulates intracellular pH critical for biomineralization in the sea urchin embryo**

Marian Y. Hu^1*^; Jia-Jiun Yan^1,2^; Inga Petersen^1^; Nina Himmerkus^1^; Markus Bleich^1^; Meike Stumpp^3^

^1^Institute of Physiology, Christian-Albrechts University of Kiel, 24118 Kiel, Germany; ^2^Institute of Cellular and Organismic Biology, Academia Sinica 115 Taipei, Taiwan; ^3^Comparative Immunobiology, Institute of Zoology, Christian-Albrechts University of Kiel, 24118 Kiel, Germany

*To whom correspondance should be addressed

**Table S1** List of species and gene accession numbers of sequences used for phylogenetic analysis of Slc4 family transporters.

| Organism | Gene Name | Gene ID |  | Organism | Gene Name | Gene ID |
| --- | --- | --- | --- | --- | --- | --- |
| Human | SLC4A1 | ENSG00000004939 |  | Cave fish | slc4a8 | ENSAMXG00000002165 |
| Human | SLC4A2 | ENSG00000164889 |  | Cave fish | slc4a10a | ENSAMXG00000002997 |
| Human | SLC4A3 | ENSG00000114923 |  | Cave fish | slc4a10b | ENSAMXG00000010002 |
| Human | SLC4A4 | ENSG00000080493 |  | Cave fish | slc4a11 | ENSAMXG00000012299 |
| Human | SLC4A5 | ENSG00000188687 |  | Cave fish | SLC4A11 | ENSAMXG00000011585 |
| Human | SLC4A7 | ENSG00000033867 |  | Zebrafish | slc4a1a | ENSDARG00000012881 |
| Human | SLC4A8 | ENSG00000050438 |  | Zebrafish | slc4a1b | ENSDARG00000024560 |
| Human | SLC4A9 | ENSG00000113073 |  | Zebrafish | slc4a2a | ENSDARG00000028173 |
| Human | SLC4A10 | ENSG00000144290 |  | Zebrafish | slc4a2b | ENSDARG00000052330 |
| Human | SLC4A11 | ENSG00000088836 |  | Zebrafish | slc4a4a | ENSDARG00000013730 |
| Mouse | Slc4a1 | ENSMUSG00000006574 |  | Zebrafish | slc4a4b | ENSDARG00000044808 |
| Mouse | Slc4a2 | ENSMUSG00000028962 |  | Zebrafish | slc4a5 | ENSDARG00000104387 |
| Mouse | Slc4a3 | ENSMUSG00000006576 |  | Zebrafish | SLC4A5 | ENSDARG00000002771 |
| Mouse | Slc4a4 | ENSMUSG00000060961 |  | Zebrafish | slc4a7 | ENSDARG00000073952 |
| Mouse | Slc4a5 | ENSMUSG00000068323 |  | Zebrafish | slc4a8 | ENSDARG00000015531 |
| Mouse | Slc4a7 | ENSMUSG00000021733 |  | Zebrafish | slc4a10a | ENSDARG00000063133 |
| Mouse | Slc4a8 | ENSMUSG00000023032 |  | Zebrafish | slc4a10b | ENSDARG00000060303 |
| Mouse | Slc4a9 | ENSMUSG00000024485 |  | Zebrafish | slc4a11 | ENSDARG00000075532 |
| Mouse | Slc4a10 | ENSMUSG00000026904 |  | Medaka | slc4a1a | ENSORLG00000002557 |
| Mouse | Slc4a11 | ENSMUSG00000074796 |  | Medaka | slc4a1b | ENSORLG00000005495 |
| Chicken | SLC4A1 | ENSGALG00000039978 |  | Medaka | slc4a2a | ENSORLG00000006900 |
| Chicken | SLC4A2 | ENSGALG00000034284 |  | Medaka | slc4a2b | ENSORLG00000012164 |
| Chicken | SLC4A3 | ENSGALG00000011224 |  | Medaka | slc4a4a | ENSORLG00000019992 |
| Chicken | SLC4A4 | ENSGALG00000031105 |  | Medaka | slc4a4b | ENSORLG00000003072 |
| Chicken | SLC4A5 | ENSGALG00000032146 |  | Medaka | slc4a5 | ENSORLG00000001244 |
| Chicken | SLC4A7 | ENSGALG00000011405 |  | Medaka | slc4a7-1 | ENSORLG00000002385 |
| Chicken | SLC4A8 | ENSGALG00000031274 |  | Medaka | slc4a7-2 | ENSORLG00000017022 |
| Chicken | SLC4A10 | ENSGALG00000030032 |  | Medaka | slc4a8 | ENSORLG00000019397 |
| Chicken | SLC4A11 | ENSGALG00000016017 |  | Medaka | SLC4A10 | ENSORLG00000016849 |
| Flycatcher | SLC4A1 | ENSFALG00000004869 |  | Medaka | slc4a10a | ENSORLG00000002809 |
| Flycatcher | SLC4A2 | ENSFALG00000013084 |  | Medaka | SLC4A11 | ENSORLG00000000723 |
| Flycatcher | SLC4A3 | ENSFALG00000004334 |  | Stickleback | slc4a1a | ENSGACG00000009622 |
| Flycatcher | SLC4A4 | ENSFALG00000008362 |  | Stickleback | slc4a1b | ENSGACG00000007018 |
| Flycatcher | SLC4A5 | ENSFALG00000000284 |  | Stickleback | slc4a2b | ENSGACG00000004664 |
| Flycatcher | SLC4A7 | ENSFALG00000009182 |  | Stickleback | SLC4A3 | ENSGACG00000002501 |
| Flycatcher | SLC4A8 | ENSFALG00000004666 |  | Stickleback | slc4a4a | ENSGACG00000014471 |
| Flycatcher | SLC4A9 | ENSFALG00000009337 |  | Stickleback | slc4a4b | ENSGACG00000015864 |
| Flycatcher | SLC4A10 | ENSFALG00000000696 |  | Stickleback | slc4a7 | ENSGACG00000003175 |
| Flycatcher | SLC4A11 | ENSFALG00000006547 |  | Stickleback | slc4a8 | ENSGACG00000000663 |
| Anole lizard | SLC4A1 | ENSACAG00000017228 |  | Stickleback | SLC4A10 | ENSGACG00000005612 |
| Anole lizard | SLC4A2 | ENSACAG00000005372 |  | Stickleback | slc4a10a | ENSGACG00000013867 |
| Anole lizard | SLC4A3 | ENSACAG00000014380 |  | Stickleback | slc4a11 | ENSGACG00000004484 |
| Anole lizard | SLC4A4 | ENSACAG00000013007 |  | Stickleback | SLC4A11 | ENSGACG00000017515 |
| Anole lizard | SLC4A7 | ENSACAG00000013876 |  | Spotted gar | slc4a1a | ENSLOCG00000012065 |
| Anole lizard | SLC4A9 | ENSACAG00000013391 |  | Spotted gar | slc4a2b | ENSLOCG00000010245 |
| Anole lizard | SLC4A10 | ENSACAG00000014632 |  | Spotted gar | SLC4A3 | ENSLOCG00000009239 |
| Anole lizard | SLC4A11 | ENSACAG00000003941 |  | Spotted gar | slc4a4a | ENSLOCG00000011541 |
| Xenopus | slc4a1 | ENSXETG00000007364 |  | Spotted gar | slc4a7 | ENSLOCG00000002412 |
| Xenopus | slc4a3 | ENSXETG00000007160 |  | Spotted gar | slc4a8 | ENSLOCG00000006747 |
| Xenopus | slc4a4 | ENSXETG00000020880 |  | Spotted gar | slc4a10a | ENSLOCG00000008549 |
| Xenopus | slc4a5 | ENSXETG00000012098 |  | Spotted gar | slc4a11 | ENSLOCG00000000878 |
| Xenopus | slc4a7 | ENSXETG00000019911 |  | Elephant Shark | SLC4A2 | SINCAMT00000010046 |
| Xenopus | slc4a8 | ENSXETG00000014124 |  | Elephant Shark | slc4a2b | SINCAMT00000018338 |
| Xenopus | slc4a9 | ENSXETG00000018334 |  | Elephant Shark | SLC4A3 | SINCAMT00000020312 |
| Xenopus | slc4a10 | ENSXETG00000016198 |  | Elephant Shark | SLC4A4-1 | SINCAMT00000005234 |
| Xenopus | slc4a11 | ENSXETG00000014645 |  | Elephant Shark | SLC4A4-2 | SINCAMT00000016168 |
| Coelacanth | SLC4A1 | ENSLACG00000016946 |  | Elephant Shark | SLC4A7 | SINCAMT00000023566 |
| Coelacanth | SLC4A2 | ENSLACG00000005985 |  | Elephant Shark | SLC4A10 | SINCAMT00000000269 |
| Coelacanth | SLC4A3 | ENSLACG00000014086 |  | Elephant Shark | SLC4A11 | SINCAMT00000018738 |
| Coelacanth | SLC4A5 | ENSLACG00000008541 |  | Ciona | slc4a2 | ENSCSAVG00000009036 |
| Coelacanth | SLC4A7 | ENSLACG00000005265 |  | Ciona | slc4a7 | ENSCSAVG00000006020 |
| Coelacanth | SLC4A8 | ENSLACG00000018933 |  | Ciona | slc4a11 | ENSCSAVG00000004447 |
| Coelacanth | SLC4A10 | ENSLACG00000002155 |  | Ciona | SLC4A11 | SINCSAVG00000005491 |
| Coelacanth | SLC4A11 | ENSLACG00000010657 |  | Lamprey | slc4a2 | ENSPMAG00000000950 |
| Cave fish | slc4a1a | ENSAMXG00000015300 |  | Lamprey | SLC4A3-1 | ENSPMAG00000002796 |
| Cave fish | slc4a1b | ENSAMXG00000012104 |  | Lamprey | SLC4A3-2 | ENSPMAG00000006912 |
| Cave fish | slc4a2a | ENSAMXG00000012634 |  | Lamprey | slc4a7 | ENSPMAG00000002081 |
| Cave fish | slc4a2b | ENSAMXG00000000548 |  | Lamprey | slc4a11 | ENSPMAG00000007011 |
| Cave fish | SLC4A3 | ENSAMXG00000018140 |  | Sea Urchin | Sp-Slc4a2 | SPU_008934 |
| Cave fish | slc4a4a | ENSAMXG00000012301 |  | Sea Urchin | Sp-Slc4a2_1 | SPU_013529 |
| Cave fish | slc4a4b | ENSAMXG00000013324 |  | Sea Urchin | Sp-Slc4a3L | SPU_018701 |
| Cave fish | slc4a7 | ENSAMXG00000007976 |  | Sea Urchin | Sp-Slc4a10 | SPU_025515 |
| Cave fish | SLC4A7 | ENSAMXG00000005426 |  | Sea Urchin | Sp-Slc4a11 | SPU_001669 |

**Table S2** pH regulatory parameters of primary mesenchyme cells in control and *SpSlc4a10* morphants along the ammonia pulse experiment. Intracellular buffercapacitiy (ß) was calculated using the equation: ß = ∆[NH^4+^]/∆pH_i_

| **Treatment** | pH_i_ start | dpH dt^-1^ | pH _pre_ | pH _post_ | ß, mol l^-1^ pH unit^-1^ |
| --- | --- | --- | --- | --- | --- |
| Control | 6.87 ± 0.13 | 0.14 ± 0.05 | 7.22 ± 0.18 | 5.88 ± 0.20 | 29.83 ± 18.16 |
| Morphant | 6.66 ± 0.17* | 0.047 ± 0.02* | 7.06 ± 0.19 | 6.06 ± 0.06 | 39.11 ± 17.16 |

**Table S3** Seawater physico-chemical parameters monitored during the pH manipulation experiment. Parameters measured include Salinity, Temperature, pH (NBS scale), and total dissolved inorganic carbon (C*_T_*). pH_NBS_ and C*_T_* were used to calculate the carbonate system, including *p*CO_2_, total alkalinity (A*_T_*) and the satuation states for calcite (Ω_Ca_) and aragonite (Ω_Ar_).

| **Treatment (pH/pCO_2_)** | **Salinity** | **Temp (°C)** | **pHNBS** | **pCO2 (µatm)** | **DIC (µM)** | **AT (µM)** | **ΩCa** | **ΩAr** |
| --- | --- | --- | --- | --- | --- | --- | --- | --- |
| **pH 8.1 (^˷^400 µatm)** | 34.68 ± 0.12 | 15.66 ± 0.04 | 8.14 ± 0.007 | 476.2 ± 9.0 | 2337.6 ± 4.9 | 2555.4 ±2.9 | 3.9 ± 0.05 | 2.5 ± 0.03 |
| **pH 7.7 (^˷^1400 µatm)** | 34.30 ± 0.04 | 15.65 ± 0.05 | 7.69 ± 0.008 | 1391.9 ±40.4 | 2414.9 ± 39.6 | 2455.7 ±39.2 | 1.5 ±0.03 | 0.9 ± 0.02 |
| **pH 7.5 (^˷^2000 µatm)** | 34.48 ± 0.19 | 15.69 ± 0.04 | 7.53 ± 0.009 | 2071.4 ± 79.3 | 2445.9 ± 40.5 | 2432.2 ± 36.9 | 1.0 ±0.01 | 0.7 ± 0.01 |

**Table S4** List of primers used for molecular cloning

| Gene Name |  | Primer |
| --- | --- | --- |
| *SpSlc4a10 (NBC)* | F | TACATTCAGGCCTACCCGCGCA |
|  | R | GGGAGGGGTGTTGGGGCATCTA |
| *SpSlc9a2 (NHE)* | F | GCTTTCTCGTTGGTGGCGCTCT |
|  | R | ACACCCCCAGGTTCATTCCACCA |
| Sp*ATP1a3 (NKA)* | F | CTCTGGAGAAACTCTGCTTT |
|  | R | CTACGATGGCACCAGCAC |

**Table S5** List of primers used for qPCR

| Gene Name |  | Primer |
| --- | --- | --- |
| *NBC* | F | GTTCTTGTTCTCTTGCGCCTC |
|  | R | AGCCAGGAAAGCCATGAAGAC |
| *NKA* | F | TTCTACCCCGTACCAGAGACCA |
|  | R | AGGGTGGAAGTTGAGTGGATGG |
| *NHE* | F | AGGAGAAACCTCTGGCAGAGCG |
|  | R | TGGGCATCACCACTAACGTCCA |
| *z12* | F | ACGGCAGCCATCTTAAGGTCCA |
|  | R | TCATGTGCTTCTTGACGCTGGC |
